# An Optimized Analytical Method for the Simultaneous Detection of Iodoform, Iodoacetic Acid, and Other Trihalomethanes and Haloacetic Acids in Drinking Water

**DOI:** 10.1371/journal.pone.0060858

**Published:** 2013-04-16

**Authors:** Xiaolin Liu, Xiao Wei, Weiwei Zheng, Songhui Jiang, Michael R. Templeton, Gengsheng He, Weidong Qu

**Affiliations:** 1 Key Laboratory of Public Health and Safety, Ministry of Education, Department of Environment Health, School of Public Health, Fudan University, Shanghai, China; 2 Key Laboratory of Public Health and Safety, Ministry of Education, Department of Nutrition and Food Hygiene, Fudan University, Shanghai, China; 3 Department of Civil and Environmental Engineering, Imperial College London, London, United Kingdom; National University of Singapore, Singapore

## Abstract

An optimized method is presented using liquid-liquid extraction and derivatization for the extraction of iodoacetic acid (IAA) and other haloacetic acids (HAA_9_) and direct extraction of iodoform (IF) and other trihalomethanes (THM_4_) from drinking water, followed by detection by gas chromatography with electron capture detection (GC-ECD). A Doehlert experimental design was performed to determine the optimum conditions for the five most significant factors in the derivatization step: namely, the volume and concentration of acidic methanol (optimized values  = 15%, 1 mL), the volume and concentration of Na_2_SO_4_ solution (129 g/L, 8.5 mL), and the volume of saturated NaHCO_3_ solution (1 mL). Also, derivatization time and temperature were optimized by a two-variable Doehlert design, resulting in the following optimized parameters: an extraction time of 11 minutes for IF and THM_4_ and 14 minutes for IAA and HAA_9_; mass of anhydrous Na_2_SO_4_ of 4 g for IF and THM_4_ and 16 g for IAA and HAA_9_; derivatization time of 160 min and temperature at 40°C. Under optimal conditions, the optimized procedure achieves excellent linearity (R^2^ ranges 0.9990–0.9998), low detection limits (0.0008–0.2 µg/L), low quantification limits (0.008–0.4 µg/L), and good recovery (86.6%–106.3%). Intra- and inter-day precision were less than 8.9% and 8.8%, respectively. The method was validated by applying it to the analysis of raw, flocculated, settled, and finished waters collected from a water treatment plant in China.

## Introduction

Widespread drinking water disinfection was one of the most significant public health advances of the 20^th^ century. However, chemical disinfection can form disinfection by-products (DBPs), some of which are known to exhibit cytotoxicity [1]–[3], genotoxicity [2], mutagenicity [3], [4], carcinogenicity [5], [6] and reproductive and developmental toxicity [7], [8]. Population-based epidemiological studies suggest that DBPs are associated with an increased risk of bladder cancer and colon cancer as well as premature birth and stillbirth [9], [10]. Given the potential health risks associated with DBPs, many countries have regulated some DBP groups.

Over 600 DBPs have been identified to-date [11]. The trihalomethanes (THMs) and haloacetic acids (HAAs) are the two major classes of DBPs which are regulated in many countries [12], however studies have indicated that some unregulated DBPs are significantly more cytotoxic and genotoxic than the currently regulated DBPs [11], [13]–[15]. Iodoform (IF) and iodoacetic acid (IAA) are two recently identified DBPs, belonging to the groups of THMs and HAAs, respectively, but they are not currently regulated in any country. Studies show that iodine-containing DBPs have greater toxicity than their chlorine-containing and bromine-containing analogues [16]. IF is the most toxic THM and also influences water odor [17], while IAA has the strongest genotoxicity of all the haloacetic acids [16].

Several methods have been developed to determine THM_4_ and HAA_9_, but these methods may not be suitable for the simultaneous determination of IF and IAA. Because the concentrations of IF and IAA in drinking water are typically very low, in the nanogram per litre range, they cannot be easily detected together with the four regulated THMs (THM_4_) and nine commonly regulated HAAs (HAA_9_), which usually are found at higher concentrations in tap waters (micrograms per litre) and are analyzed by methods which are not sensitive enough to detect IF and IAA [18]–[20].

The existing sample pre-treatment procedure options are liquid-liquid extraction (LLE), liquid-liquid microextraction (LLME), solid phase extraction (SPE), solid phase microextraction (SPME), fiber membrane extraction, and head-space extraction (HS) [21]–[24]. However IF and THM_4_ are poorly recovered using SPE because of their volatility. HAA_9_ require derivatization when using gas chromatography for detection. Some derivatization agents, such as diazomethane, are suspected carcinogens [25] while other agents, such as bromopentafluorobenzene (PFBBr), can form unstable derivatives and interfere with the detection of brominated compounds [24], [26]. Gas chromatography (GC) with electron capture detection (GC-ECD) [27]–[28], GC mass spectrometry (GC-MS) [23]–[24], [29], high performance liquid chromatography with mass spectrometry (HPLC-MS) [30]–[31], ultra-performance liquid chromatography with mass spectrometry (UPLC-MS) [30], ion chromatography (IC) [32] and capillary electrophoresis (CE) [19], [33] have been demonstrated as methods for the analysis of THM_4_ and HAA_9_. For IF and THM_4,_ which are volatile DBPs, GC is a better option than HPLC because of higher sensitivity and selectivity. For IAA and HAA_9_, LC-MS and IC-MS can greatly reduce the pre-processing time and reduce the loss of target analytes by avoiding the need for derivatization. However, acidic buffers or ion-pairing reagents are usually required for these two methods to increase the retention of HAAs, which lead to suppression of the ionization of HAAs in the electrospray ionization source [19]. CE methods achieve better separation but the injection volume is only in the range of nanolitres, resulting in the higher detection limits than from the IC and HPLC methods [19]. Furthermore, IAA, IF, THM_4_ and HAA_9_ all include one or more halogen elements, which makes ECD appropriate, given that ECD achieves higher selectivity and sensitivity for halogenated compounds than MS and other detectors. Therefore, the focus of this study was to establish an optimized GC-ECD method for the simultaneous measurement of IF and IAA along with the other THMs and HAAs.

Traditionally, optimization in analytical chemistry has been carried out by monitoring the influence of one factor at a time while other factors are held constant. However this optimization approach does not consider the potential interaction amongst the variables studied and increases the number of experiments required [34]. Response surface methodologies, such as two-level factorial, central composite, Box-Behnken, and Doehlert designs, can optimize two or more factors simultaneously. These techniques account for interactions between factors and reduce the number of experiments required. In particular, Doehlert design has been argued to be one of the most efficient of these methods and has been applied to a number of method optimization problems previously [35], [36].

The objective of the present study was to optimize the extraction time, volumes of extraction agents, derivatization time and temperature and concentration and volume of derivatization agents using Doehlert design and to establish a method for the simultaneous analysis of IAA and HAA_9_ and IF and THM_4_. The results suggest that the method achieves low detection limits, high recovery and minimizes organic solvent usage, and it uses GC analysis instead of more expensive analytical instruments.

## Materials and Methods

### Ethics Statement

This study was part of a non-profit project supported by the Chinese Ministry of Science & Technology. All necessary permits were obtained for the described field studies and approved by the Shanghai Municipal Water Affairs Bureau (2008ZX07421-004).The sampling location was not privately owned or protected in any way, and the field studies did not involve endangered or protected species.

### Reagents

Methyl tert-butyl ether (MTBE) (99.9%) which was used as an extraction solvent was purchased from TEDIA (St. Louis, MO, USA). Methanol was of HPLC grade and obtained from Merck KGaA (Darmstadt, Germany). Inorganic reagents (H_2_KO_4_P, HNa_2_O_4_P, NH_4_Cl, NaHCO_3_) were of analytical grade and were all obtained from Sigma-Aldrich (St. Louis, MO, USA). Anhydrous sodium sulfate was of analytical standard and purchased from Fluka (St. Louis, MO, USA). Analytical grade sulfuric acid which was used as a pH regulator was purchased from Sinapharm Chemical Reagent Co., Ltd (Shanghai, China). A haloacetic acids mix (including chloroacetic acid (CAA), bromoacetic acid (BAA), dichloroacetic acid (DCAA), trichloroacetic acid (TCAA), bromochloroaceic acid (BCAA), dibromoacetic acid (DBAA), bromodichloroacetic acid (BDCAA), chlorodibromoacetic acid (CDBAA), and tribromoacetic acid (TBAA)) was used as a stock solution (stored in 2×10^6^ µg/L in MTBE) and was obtained from Supelco (Bellefonte, PA, USA). IAA (99%) and bromoform (BF) (97.9%) were purchased from Sigma-Aldrich (St. Louis, MO, USA) as neat standards. Dibromochloromethane (DBCM) (98.7%) and Bromodichloromethane (BDCM) (99%) were also neat standards and obtained from Fluka (St. Louis, MO, USA). Chloroform (CF) (98.6%) and IF (99%) were obtained from Chem Service and Aldrich, respectively. Bromofluorobenzene (99%) and 1, 2-dibromopropane (97%), which were used as internal standards in the analysis of THMs and HAAs, were obtained from Aldrich (St. Louis, MO, USA). Ultra-pure water which was used in the experiments was processed through a Milli-Q water system (Millipore, Bedford, MA, USA).

### Preparation of Standard Solutions

The HAA mixture standard was commercially available. A stock standard solution (1.0×10^6 ^µg/L) of each THM and IF was prepared in MTBE [28]. A stock standard solution (1.0×10^6 ^µg/L) of IAA was prepared in ultra-pure water. Primary dilution standards (1.0×10^5 ^µg/L) containing a mixture of four THMs were prepared by dilution of an appropriate quantity of stock solutions with MTBE. HAAs mix and IF primary dilution standards were also prepared in MTBE while IAA was prepared in ultra-pure water. Second dilution standards of IAA (1.0×10^3 ^µg/L) and IF (1.0×10^4 ^µg/L) which were used as working standard solutions were all prepared in MTBE. Concentrations of bromofluorobenzene and 1, 2-dibromopropane stock standard solutions were 1.0×10^7 ^µg/L and 1.0×10^6 ^µg/L, respectively. MTBE containing 1, 2-dibromopropane (120 µg/L) was used as the extraction solvent of IAA and HAA_9_.

### Sample Preparation

Parameters affecting the extraction and derivatization procedure were optimized. For IF and THM_4_, a single-factor experimental design with multiple levels was developed to optimize the MTBE volume, while a Doehlert design was applied to two other parameters, inorganic salt (Na_2_SO_4_) and extraction time (Table S1). Under the optimal conditions, 3.0 mL MTBE and 4 g Na_2_SO_4_ was added to 50 mL of water sample. The mixture was extracted for 11 minutes. The vial was inverted for five minutes and allowed the water and MTBE phases to separate. The MTBE phase was transferred to an autosampler vial and stored at −20°C for confirmation analysis.

For IAA and HAA_9_, optimization of the extraction procedure was similar as for IF and THM_4_ (Table S2). Derivatization time and temperature was also optimized using two-variable Doehlert design (Table S3) while other parameters of the derivatization process, i.e. the concentration and volume of acidic methanol and Na_2_SO_4_ solution and the volume of NaHCO_3_ solution, were optimized using 2^5^ factorial design and a Doehlert design (Table S4). After the optimization, the optimal conditions for IAA and HAA_9_ were as follows: 2 mL concentrated sulfuric acid was added to 40 mL water sample and then 16 g of Na_2_SO_4_. The water sample was shaken vigorously by hand until all Na_2_SO_4_ was dissolved. Next, 3.0 mL of MTBE with internal standard was added. The sample was shaken vigorously for 14 minutes and the phases were allowed to separate for five minutes. Then 2 mL of the upper MTBE layer was transferred to a 15-mL graduated conical centrifuge tube and 1 mL of 15% acidic methanol was added to each centrifuge tube. After sealing, the tubes were placed in a water bath at 40°C and heated for 160 minutes. The tubes were then removed from the water bath and cooled to room temperature. A volume of 8.5 mL of a 129 g/L Na_2_SO_4_ solution was added to each centrifuge tube and the lower layer was discarded. Then 1 mL of saturated NaHCO_3_ solution was added and the upper ether layer was transferred to an autosampler vial. Extracts were stored at −20°C for confirmation analysis.

### GC-ECD Analysis

Analysis was carried out using a Shimadzu-QP2010 (Shimadzu, Japan) GC equipped with a split/split-less injector and an electron capture detector (ECD, 63Ni). Compounds were separated on a fused silica DB-1MS capillary column (30 m×0.25 mm×0.25 µm film thickness) (Agilent Technologies, Palo Alto, CA, USA). For THM_4_ and IF, helium was used as the carrier gas and nitrogen was used as the makeup gas at flow rates of 1.4 and 30 mL/min, respectively. The GC was operated in split-less mode with the injector temperature at 230°C. The oven temperature was maintained at 35°C for 15 min, and then programmed at 25°C/min to 145°C held for 3 min, and finally 35°C/min to 240°C which was held for 5 min. The separated species were measured by ECD held at 260°C. For HAA_9_ and IAA, the flow rates of carrier and makeup gas, injection mode and injector temperature were the same as for THM_4_ and IF. The oven temperature was maintained at 40°C for 10 min, and then programmed at 10°C/min to 85°C, and finally 30°C/min to 205°C which was held for 5 min. The separated species were measured by ECD held at 260°C.

### Statistics

All the data derived from the Doehlert experimental design were analyzed statistically using Design Expert 8.0.6.1 software (Stat-Ease, Minneapolis, MN, USA). Data obtained from single factor and two- or three-level statistical tests were analyzed using SPSS 16.0 software (IBM Corporation, Armonk, New York, USA).

For data of Doehlert design that complied with the normal distribution and homogeneity of variance, a one-way ANOVA was used and gave P values and the value of the lack of fit. For single factor and two- or three-level data that complied with the normal distribution and homogeneity of variance, a t-test (two-level) or ANOVA (three-level) was carried out followed by Dunnett’s Multiple Comparison test with the significance level set at *P*<0.05. For data that did not complied with the normal distribution and homogeneity of variance, a t-test (two-level) or a Wilcoxon rank test was used.

## Results and Discussion

### Optimization for the Derivatization Conditions of IAA and HAA_9_

Parameters affecting the derivatization process included derivatization time and temperature, the volume of organic solvent (MTBE), the concentration and volume of Na_2_SO_4_ solution and acidic methanol, and the volume of saturated NaHCO_3_ solution [25]. Among these parameters, derivatization time and temperature were expected to be two especially critical factors [27]. Na_2_SO_4_ solution could increase the ionic strength of the aqueous phase and thus drive the target analytes into the organic phase; however no previous studies reported the best concentration and volume of Na_2_SO_4_ solution to achieve optimal separation. Therefore, derivatizationg time and temperature were studied by means of a Doehlert design, while the other parameters of the derivatization process (concentration and volume of Na_2_SO_4_ solution and acidic methanol, volume of saturated NaHCO_3_ solution) were optimized using two sequential experimental designs: a fractional factorial 2^5^ design involving 32 experiments was applied to establish the relative influence of the factors and a Doehlert experimental design was developed to study the most significant factors. The volume of organic solvent (MTBE) was optimized by a single factor with three-level statistical analysis.

### Derivatization Temperature and Time

Derivatization temperature and time were two critical factors affecting derivatization efficiency and one study suggested that an increase of these two factors could increase the derivatization efficiency of HAA_9_, especially trihaloacetic acids (TXAAs) [25]. However, an unlimited increase of derivatization temperature and time leads to lengthening the operation time and excessively high temperature may result in the loss of the derivatives because of the volatility of MTBE. Moreover, previous research did not consider IAA detection and the conditions may not be suitable for IAA determination. In this study, a Doehlert design was used to optimize derivatization temperature and time, with the peak area of each analyte being the response variable (*Y*).

**Figure 1:**
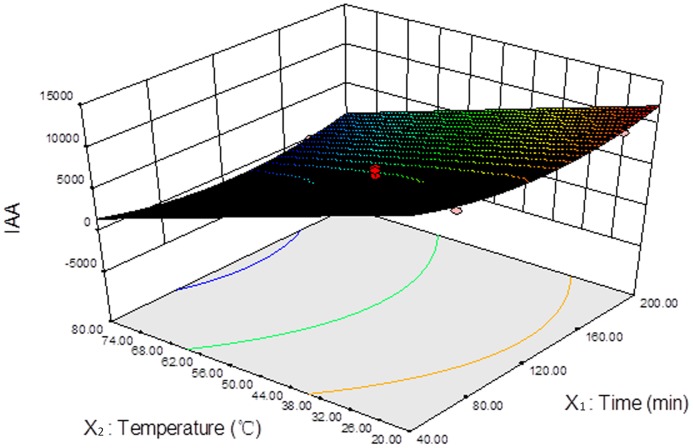
3D response surface of IAA for optimization of derivatization temperature and time. X_1_ was derivatization time (min), X_2_ was temperature (°C) and *Y* was the peak area of IAA.

*P* values of all the models and coefficients were less than 0.05 and *P* values of the lack of fit were greater than 0.05, which meant that the models and coefficients in this experiment were statistically significant. A 3D response surface figure obtained from the software demonstrated that the impact of the derivatization temperature on the efficiency of IAA derivatization was significantly greater than the impact of derivatization time (Fig. 1). There was a small interaction between derivatization time and temperature and these two factors had negative effects on derivatization efficiency. Low temperature and short time enhanced the generation of IAA derivative. On the basis of these responses (peak area counts), a second-order model suitable for predicting the responses in all experimental regions was obtained: *Y* = +5166.96−496.62*X*_1_−5773.62*X*_2_+337.05*X*_1_ *X*_2_+2131.56*X*_1_^2^ where *Y* was the IAA peak area, and *X*_1_ and *X*_2_ corresponded to derivatization time and temperature, respectively.

However, decreasing the derivatization time and temperature affects the derivatization efficiency of dihaloacetic acids (DXAAs) and trihaloacetic acids (TXAAs). The models and 3D response surfaces (Table S5) indicated that the derivatization efficiency of CAA and BAA decreased with increasing temperature while that of DXAAs and TXAAs exhibited a bell-shaped curve in relation to temperature. DXAAs and TXAAs had the highest derivatization efficiency between 40°C and 50°C; lower temperatures affected the reaction rate while higher temperatures increased the loss of the analytes via MTBE volatilization. Derivatization time had no effect on the derivatization efficiency of BAA, and showed a negative effect on that of CAA; however, the interaction between time and temperature weakened this negative effect. Derivatization efficiency of DXAAs and TXAAs increased with increasing derivatization time. The results were consistent with a previous study, wherein higher derivatization efficiency of HAA_9_, especially TXAAs, was achieved at longer derivatization time and higher temperature [25].

The application of the Derringer function [34], based on constructing a desirability function (obtained by calculating the individual desirability (d_i_) and overall desirability (D), which is the weighted geometric average of the d_i_) for each individual response, indicated the presence of an optimal result, which corresponded to 160 min of derivatization time and 40°C temperature.

### The Volume of Organic Solvent (MTBE)

In order to investigate the effect of organic solvent volume on derivatization efficiency, a single factor experiment with three-level comparative analysis was used with MTBE volumes of 2, 3 and 4 mL. The response variable was the recovery of each HAA compound. The P value of normality and homogeneity of variance test was greater than 0.05, which suggested the data could be analyzed by one-way ANOVA. The results indicated that the derivatization efficiency was statistically different when using different MTBE volumes (Table 1). However, the recovery was acceptable, in the range of 80%–120%, for all the MTBE volumes, so the volume of MTBE was not considered a key factor affecting derivatization efficiency. However, peak areas of HAA_9_ and IAA decreased with increase of the volume of MTBE, which may influence the IAA and HAA_9_ detection. Considering the above results, 2 mL MTBE was selected as the optimal volume.

**Table 1:** Recovery of IAA and HAA_9_ in different volumes of derivatization solvent (MTBE) (%, *Mean ± SD*, n = 10).

MTBE(mL)	IAA	CAA	BAA	DCAA	BCAA	DBAA	TCAA	BDCAA	CDBAA	TBAA
2	97.8±9.8*	112.6±5.3*	110.0±6.7*	110.9±3.7*	109.4±3.0*	110.4±3.4*	110.8±4.6	104.0±5.0*	108.7±7.6	106.5±8.0
3	80.0±2.9	94.7±4.0*	93.7±5.4	91.2±4.4*	91.2±3.8*	96.6±4.7*	95.6±4.2	113.6±3.8*	108.3±7.3	103.1±6.3
4	107.6±5.7*	100.6±6.2*	97.2±6.9	97.9±4.5*	98.7±4.0*	104.9±7.3*	100.4±5.7	84.0±6.5*	83.1±6.1*	104.4±6.5
*F* value	23.4	21.9	15.8	52.9	55.4	19.24	25.8	30.6	15.6	0.387
*P* value	0.000	0.000	0.000	0.000	0.000	0.000	0.000	0.000	0.000	0.688

* *P*<0.05 is significant statistically.

### Screening the Derivatization Reagents by a 2^5^ Factorial Design

The reagents affecting the derivatization procedure were evaluated by a two-level factorial design. This consisted of testing combinations of the levels of the different factors and their interactions. Two levels, expressed as coded values (+1) and (−1), were considered for each of the five factors (concentration and volume of Na_2_SO_4_ solution and acidic methanol, volume of saturated NaHCO_3_ solution). Under these conditions, a complete factorial design required 32 experiments. All the data conformed to a normal distribution with homogeneity of variance, so a one-way ANOVA analysis suggested that these five factors all influenced derivatization efficiency of IAA and HAA_9_ and had interactions. Therefore, these five factors required a further optimization.

### Optimization of Derivatization Reagents by a Doehlert Design

**Figure 2:**
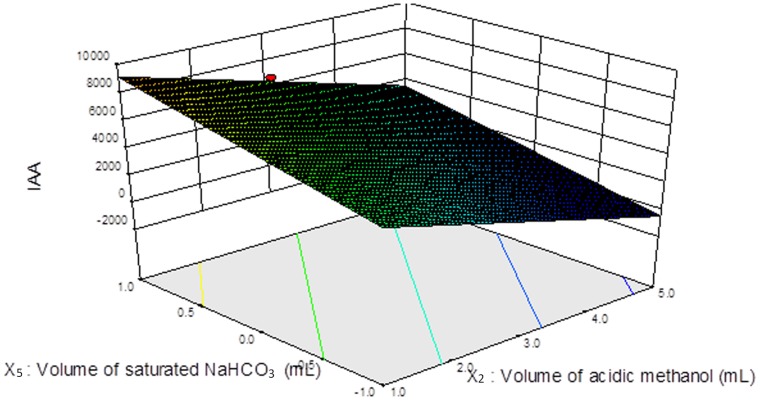
3D response surface of IAA: volume of acidic methanol versus volume of saturated NaHCO_3_ solution. X_2_ was the volume of acidic methanol (mL), X_5_ was the volume of saturated NaHCO_3_ solution (mL) and *Y* was the peak area of IAA.

A Doehlert design with 34 experiments was applied to optimize the use of the derivatization reagents. The Design Expert software produced statistically significant models with *P* value less than 0.05 and insignificant lack of fit. It was observed that improved derivatization efficiency for IAA was obtained with high volume of acidic methanol and low volume of saturated NaHCO_3_ solution and the interactions between them were not statistically significant (Fig. 2). A fitted model was as follows: *Y* = 8503.72−1384.66*X_2_*+2022.42*X_5_*, where Y was the peak area of IAA, *X_2_* and *X_5_* were the volume of acidic methanol and saturated NaHCO_3_ solution, respectively. The volume of acidic methanol had a negative effect on CAA and BAA derivatization efficiency. Higher derivatization efficiency was obtained at higher concentration of acidic methanol. On the other hand, DBAA, TCAA and CDBAA were influenced by all five variables, which had interactions between them. Moreover, improved BDCAA and TBAA derivatization efficiency was achieved when increasing the concentration and volume of Na_2_SO_4_ and decreasing the volume of saturated NaHCO_3_ solution (Table S6).

Monohaloacetic acids (MXAAs) and DXAAs (excluding DBAA) were only affected by acidic methanol while DBAA and TXAAs were influenced by the five factors studied and had statistically significant interactions between them. This suggests something unique about the derivatization of DBAA and TXAA. The negative impact of increasing volume of acidic methanol on derivatization efficiency may result from increasing the solubility of MTBE in the water phase, which leads to the loss of derivatives [25]. Higher concentration of Na_2_SO_4_ solution could also lead to crystallization and salt crystals may adsorb the derivatives [37]. Small volume of saturated NaHCO_3_ solution may give rise to smaller peak areas of DBAA and TXAA because the NaHCO_3_ solution cannot neutralize the H_2_SO_4_ completely and the residual acid catalyzes the hydrolysis of the haloacetates, especially trihalolacetates [38].

Considering the above factors, the optimized conditions were selected as: 1 mL of 15% acidic methanol, 8.5 mL of 129 g/L Na_2_SO_4_ solution and 1 mL of saturated NaHCO_3_ solution.

### Optimization of Extraction Conditions

The volume of MTBE, dosage of Na_2_SO_4_, and extraction time affected the extraction efficiency of IAA and HAA_9_ as well as IF and THM_4_. MTBE and extraction time directly influenced the concentration of the extracts while anhydrous sodium sulfate promoted the transfer of the targets from the aqueous phase into the organic phase [27]. The volume of MTBE for IAA and HAA_9_ as well as IF and THM_4_ was studied using single factor with two or three-level statistical analysis while the amount of Na_2_SO_4_ and extraction time was explored by two-variable Doehlert design.

### Extraction Solvent: MTBE

Volumes of MTBE of 2, 3, and 4 mL were considered under the primary IF and THM_4_ pretreatment procedure conditions (Table 2). The results obtained from SPSS 16.0 indicated that the recoveries of THM_4_ were not significantly different while IF exhibited low recovery when MTBE was 2 mL. As such, 3 mL MTBE was selected as the optimal condition, to minimize solvent usage.

**Table 2:** Comparison of the IF and THM_4_ recovery in different volumes of MTBE (%, Mean ± *SD*, n = 5).

MTBE (mL)	CF	BDCM	DBCM	BF	IF
2	93.8±6.3	90.7±5.0	89.2±4.7	89.5±5.1	56.0±4.7*
3	84.2±4.2	84.0±4.9	87.4±5.7	87.6±6.2	87.2±6.1
4	91.9±7.7	89.7±6.9	91.4±6.1	94.8±7.3	82.4±5.6
*F* value	3.31	1.99	0.64	1.78	47.04
*P* value	0.072	0.179	0.544	0.211	0.000

* *P*<0.05 is significant statistically.

The results suggested that there was a statistically significant difference caused by using 3 versus 4 mL of MTBE on BAA, IAA, BCAA, BDCAA and CDBAA recovery (Table 3). However, the recovery of the above HAAs (except for IAA) ranged from 80% to 120% when MTBE was 3 and 4 mL, which was deemed acceptable in practice. Considering the recovery of IAA was only 78% in 4 mL MTBE and that the peak areas of the compounds in 3 mL MTBE were higher than when using 4 mL MTBE, 3 mL of MTBE was selected as optimal for IAA and HAA_9_ extraction.

**Table 3:** Recovery of IAA and HAA_9_ in different volumes of extraction solvent (MTBE) (%, *Mean* ± *SD*, n = 12).

MTBE(mL)	CAA	BAA	DCAA	IAA	TCAA	BCAA	DBAA	BDCAA	CDBAA	TBAA
3	102.0±4.6	95.5±6.0*	97.7±5.2	102.8±8.3*	98.2±4.7	93.3±4.6*	93.2±3.3	91.2±8.4*	97.2±6.2*	98.4±7.9
4	103.1±5.8	105.3±3.0	99.7±4.4	78.0±5.9	95.1±7.5	99.2±4.6	96.3±7.9	100.0±1.7	84.0±2.0	94.7±7.3
t/t’ value	−0.449	−4.860	−1.062	5.622	1.094	−3.213	−1.200	−2.276	6.094^a^	1.087
*P* value	0.660	0.000	0.299	0.000	0.290	0.004	0.247	0.042	0.000	0.291

* *P*<0.05 is significant statistically.

### Extraction Time and Mass of Anhydrous Sodium Sulfate

A two-factor Doehlert design was used to optimize the extraction time and mass of anhydrous sodium sulfate. Replicates at the central level of the variables were performed in order to validate the model by means of an estimate of experimental variance. The statistical analysis was same as in the investigation of derivatization temperature and time discussed above, and all data could be analyzed by ANOVA analysis. The extraction time was varied from 2 to 18 min and the mass of anhydrous sodium sulfate was ranged from 4 to 22 g for IF and THM_4_ while 10 to 26 g for IAA and HAA_9_.

**Figure 3:**
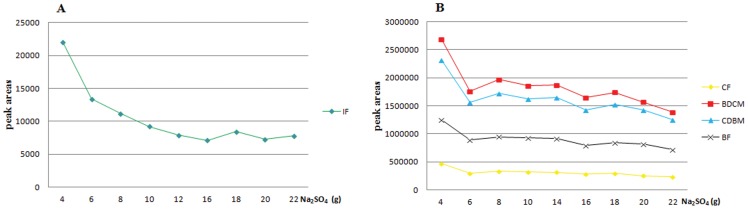
A: Peak area of IF from different amounts of anhydrous sodium sulfate. *X* and *Y* axes represent the mass of anhydrous sodium sulfate (g) and peak areas of IF, respectively. The peak areas of IF exhibited a downward trend with the increase of anhydrous sodium sulfate. B: Peak areas of THM_4_ from different amounts of anhydrous sodium sulfate. *X* and *Y* axes represent the mass of anhydrous sodium sulfate (g) and peak areas of THM_4_, respectively. The peak areas exhibited a downward trend with the increase of the anhydrous sodium sulfate and the compounds had the largest peak areas when the mass of anhydrous sodium sulfate used was 4 g.

The results indicated that the mass of anhydrous sodium sulfate had no effect on the recovery of IF and THM_4_ (*P*>0.05) while extraction time showed a positive effect on THM_4_ and no effect on IF. An extraction time of 11 min yielded the highest recovery. Considering the effect of the mass of anhydrous sodium sulfate on precipitation of MTBE extracts, we further studied the influence of the mass of anhydrous sodium sulfate on the peak areas of the analytes; the results suggested that the highest peak areas was obtained when amount of anhydrous sodium sulfate was 4 g for IF and THM_4_ (Fig. 3A and 3B).

**Figure 4:**
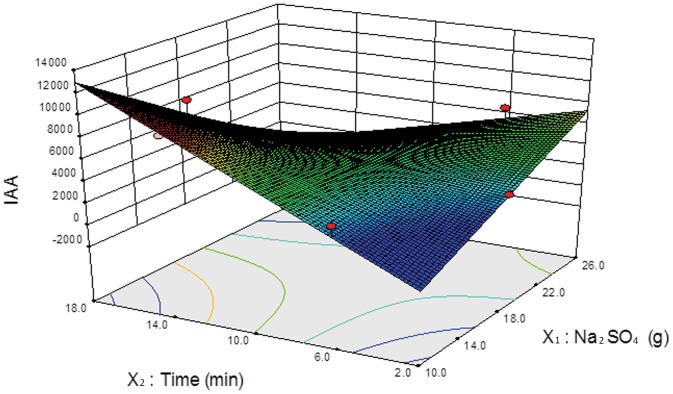
3D response surface of IAA for optimization of extraction time and mass of anhydrous sodium sulfate. X_1_ was the mass of anhydrous sodium sulfate (g), X_2_ was extraction time (min) and *Y* was the peak area of IAA.

For IAA, CDBAA and DBAA, the extraction time and mass of anhydrous sodium sulfate had a strong positive influence on the peak area of IAA (Fig. 4). Interactions of these two factors showed a negative effect. Extraction efficiency of CAA, BAA and DCAA was only related to the mass of anhydrous sodium sulfate and the relationship was not linear but rather an upward parabola. TCAA was only influenced by extraction time, showing a linear positive correlation. The optimal conditions were 14 min of extraction time and 16 g of anhydrous sodium sulfate for IAA and HAA_9_.

### Optimization of GC-ECD Conditions

The gas chromatographic separation was optimized in terms of the injection, detector and column temperature and carrier gas flow rate. Injection and detector temperature depended on the boiling point of the target analytes and the maximum temperature of the column. Column temperature and carrier gas velocity were the especially critical factors for separation of the target analytes.

**Figure 5:**
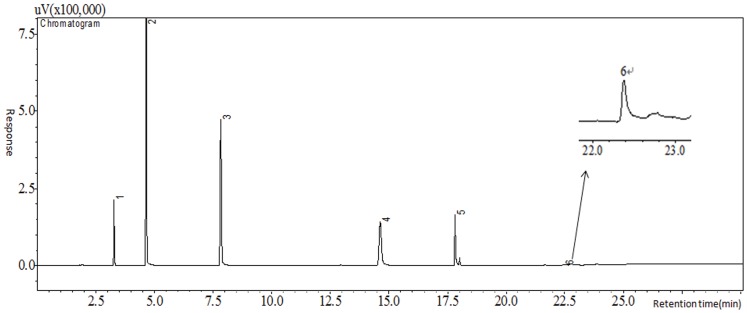
Chromatogram of IF and THM_4_. The concentration of each THM was 10 µg/L and that of IF was 1.0 µg/L. 1 stood for CF, 2 was BDCM, 3 was CDBM, 4 was BF, 5 was the internal standard (bromofluorobenzene) and 6 was IF.

**Figure 6:**
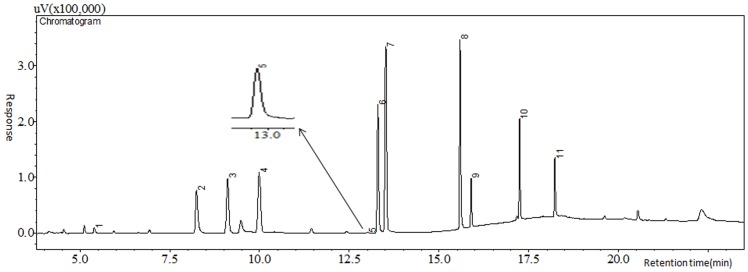
Chromatogram of IAA and HAA_9_. The concentration of each HAA was 10 µg/L and that of IAA was 1.0 µg/L. Numerals 1 to 11 represent CAA, BAA, DCAA, the internal standard (1, 2-dibromopropane), IAA, TCAA, BCAA, DBAA, BDCAA, CDBAA and TBAA, respectively.

The programmed temperature settings, including initial temperature (30–50°C with an interval of 5°C) and its hold time (5–20 min with an interval of 5 min), second and third stage of heating rate ranging from 10°C/min to 30°C/min and from 20°C/min to 35°C/min, respectively, with an interval of 5°C/min, second and third stage of hold time ranging from 0 min to 9 min with an interval of 3 min and from 0 min to 10 min with an interval of 5 min, were optimized. The results indicated that improved IF separation was achieved when the hold time of initial temperature was longer, while IAA was not sensitive to the programmed temperature. Considering both separation effect and analysis time, the optimized programmed temperature was as follows: 35°C initial held for 15 min, programmed 25°C/min to 145°C held for 3 min, then 35°C/min to 240°C held for 5 min for IF and THM_4_ (total 30.11 min) (Fig. 5), and 40°C initial held for 10 min, 10°C/min to 85°C, then 30°C/min to 205°C held for 5 min for IAA and HAA_9_ (total 23.5 min) (Fig. 6).

The carrier gas velocity was studied in the range of 0.8 to 1.6 mL/min, keeping the rest of the programmed temperature unchanged as above. When the flow rate was 0.8 mL/min or 1.6 mL/min, IF and BF could not be separated from miscellaneous peaks nearby, whereas 1.0, 1.2 and 1.4 mL/min achieved a similarly good separation. The flow rate of 1.4 mL/min allowed shorter analysis time and thinner peaks for the target analytes, so 1.4 mL/min was selected as the optimal carrier gas velocity for IF and THM_4_. For IAA, reducing the flow rate distinctly improved the separation effect but the lower rate (0.8 mL/min) led to peak tailing; therefore, the carrier gas velocity was set at 1.4 mL/min for IAA and HAA_9_.

Taking into account of the boiling point of the target analytes and the maximum temperature of the column, the injection and detector temperatures were set at 230°C and 260°C, respectively.

### Method Performance

Quality control (QC) of the new method, included laboratory and field reagent blanks, linearity range, limits of detection (LOD) and quantification (LOQ), recovery, repeatability and reproducibility, was carried out in strict compliance with the EPA requirements [27]–[28]. The linearity was tested using standard mixtures at different concentrations in the range 0.001–160 µg/L for each compound. The peak area ratio of each target compound to the internal standard was used. The range of linearity was 0.01–100 µg/L for IF and THM_4_ and 0.01–150 µg/L for IAA and HAA_9_. Correlation coefficients of the calibration curves for each compound were between 0.9982 and 0.9999, which met the quantitative requirements (Table 4). The LOD and LOQ, defined as the concentration that gave a response equivalent to three and ten times the standard deviations of the blank, respectively, were found to be between 0.0008–0.005 µg/L for LOD and 0.008–0.02 µg/L for LOQ of IF and THM_4_, and 0.004–0.2 µg/L for LOD and 0.008–0.4 µg/L for LOQ of IAA and HAA_9_. These detection limits were deemed suitable for the analysis of the low typical concentrations of these compounds in drinking water, especially for IAA and IF.

**Table 4:** Performance and validation of the LLE-GC-ECD method for IF, THM_4_ and IAA, HAA_9_.

Compound	Linearity	R^2a^	LOD	LOQ	Recovery(%,n = 6)		Repeatability (RSD, %, n = 6)			
	(µg/L)		(µg/L)	(µg/L)	Low^b^	High^c^	Intra-day^d^		Inter-day^e^	
							Low b	High^c^	Low b	High^c^
IAA	0.01–150.0	0.9990	0.004	0.01	106.3	86.6	5.3	5.0	8.8	4.4
CAA	0.5–150.0	0.9996	0.2	0.4	101.0	96.5	8.9	5.6	3.8	7.0
BAA	0.1–150.0	0.9999	0.01	0.08	99.1	99.2	7.6	4.7	4.0	3.5
DCAA	0.01–150.0	0.9994	0.006	0.008	96.2	98.8	4.5	3.4	7.7	5.2
BCAA	0.01–150.0	0.9993	0.004	0.01	94.2	98.1	8.9	5.5	7.9	7.9
DBAA	0.02–150.0	0.9999	0.004	0.02	100.4	100.0	8.0	4.2	3.4	7.0
TCAA	0.01–150.0	0.9998	0.006	0.008	104.2	96.3	4.5	7.0	9.7	6.1
BDCAA	0.1–150.0	0.9990	0.04	0.08	105.7	97.8	5.6	3.3	4.2	7.7
CDBAA	0.02–150.0	0.9990	0.01	0.02	92.4	99.9	6.7	7.1	4.9	7.7
TBAA	0.5–150.0	0.9991	0.1	0.4	92.1	92.8	6.4	6.7	2.5	4.9
IF	0.02–20.0	0.9994	0.005	0.02	98.8	95.4	2.9	1.9	6.2	6.5
CF	0.01–100.0	0.9982	0.005	0.01	98.6	100.6	5.0	6.5	4.5	3.7
BDCM	0.01–50.0	0.9996	0.001	0.008	98.0	103.3	6.2	2.8	5.0	2.5
CDBM	0.01–50.0	0.9998	0.0008	0.008	100.5	102.7	5.7	5.0	4.6	2.3
BF	0.01–50.0	0.9995	0.002	0.008	101.3	100.1	5.5	4.1	3.6	2.7
EPA^f^	–	–	–	–	80–120	80–120	≤15	≤15	≤15	≤15

a R^2^– The correlation coefficient of the standard calibration curve.

b Low means low level of compound. IAA is 0.01 µg/L and HAA_9_ is 1.0 µg/L, while IF is 0.04 µg/L and THM_4_ is 2.0 µg/L.

c High means high level of compound. IAA is 0.1 µg/L and HAA_9_ is 8.0 µg/L, while IF is 0.5 µg/L and THM_4_ is 12.0 µg/L.

d Intra-day RSD was determined by analyzing six replicates for each level on the same day.

e Inter-day RSD was performed by analyzing six replicates for 6 days.

f This line shows the quality control standards of U.S. EPA [27]–[28].

The repeatability and reproducibility of the method was assessed by inter- and intra-day RSD for n = 6 consecutive injections of a standard, containing all the target species at the level of 1.0 and 8.0 µg/L for each HAA, 0.01 and 0.1 µg/L for IAA, 2.0 and 12.0 µg/L for each THM, 0.04 and 0.5 µg/L for IF. The results (Table 4) indicated that all inter- and intra-day RSDs were below 10%, meeting the EPA requirement of RSDs below 15%. The recoveries of the method were tested by analyzing spiked samples at two levels and the results showed that the mean recoveries (n = 6) were in the range of 95.4% – 103.3% for IF and THM_4_ and 86.6%–106.3% for IAA and HAA_9_, which complied with the detection standards of EPA for THM and HAA analysis.

The optimized method presents several advantages over other methods for THM_4_ and HAA_9_ determination (Table 5). Firstly, it can simultaneously detect IAA, HAA_9_ and IF, THM_4_. Furthermore, the pre-treatment process is simple and its cost is relatively low. Organic solvent consumption is low and it uses an environment friendly extraction agent and derivative agent. GC–ECD achieves better separation than LC and is fast and relatively low cost.

**Table 5:** Comparison of different detection methods.

Detection method	Target analytes	Recovery(%)	LOD (µg/L)	Repeatability(%)	Reproducibility(%)	Technical requirements	Cost	Reference
HAAs								
LLE-GC-ECD	IAA, HAA_9_	86.8–106.3	0.0040–0.20(IAA:0.0040)	3.3–8.9	2.5–9.7	Low	Low	This study
LLE-GC-ECD	HAA_9_	92.2–128.0	0.012–0.17	0.4–4.7	–	Low	Low	[27]
HS-SPME-GC-ECD	HAA_9_	–	0.029–0.28	10.0–20.0	15.0–25.0	Middle	Middle	[39]
SDME-GC-MS	HAA_6_	82.5–97.6	0.10–1.20	5.1–8.5	8.8–12.3	High	High	[24]
SPE-LC-MS/MS	HAA_5_	60.1–102.4	0.040–0.31	2.4–6.6	3.8–14.8	High	High	[40]
LC-MS/MS	HAA_9_	80.1–108.0	0.16–8.87	<8.7	<12.0	High	High	[31]
HPLC-MS/MS	IAA, HAA_9_	–	0.18–71.50(IAA:3.21)	1.5–17.3	0.2–18.0	High	High	[30]
UPLC-MS/MS	HAA_9_	87.2–106.7	0.060–0.16	1.3–5.8	0.9–7.5	High	High	[41]
IS-PCR-IC	HAA_9_	75.9–112.0	1.40–7.80	6.2–34.6	–	Middle	Middle	[42]
PAEKI-CE-MS/MS	IAA, HAA_9_	76.0–125.0	0.013–0.12(IAA:0.013)	5.8–14.4	–	High	High	[19]
THMs								
LLE-GC-ECD	IF, THM_4_	95.4–103.3	0.00080–0.0010(IF:0.0050)	1.9–6.5	2.3–6.5	Low	Low	This study
LLE-GC-ECD	THM_4_	97.0–110.0	0.0050–0.075	0.7–1.9	–	Low	Low	[28]
DLLME-GC-microECD	THM_4_	79.0–113.0	0.050–1.30	1.0–15.5	9.2–13.1	Middle	Middle	[43]
HS-SPME-GC-µECD	THM_4_	74.7–120.9	0.057–0.32	–	–	Middle	Middle	[44]
HFLPME-GC-ECD	THM_4_	80.3–104.2	0.018–0.049	–	–	Middle	Middle	[44]
HS-GC-MS	THM_4_	86.3–90.0	0.023–0.10	–	–	High	High	[44]
HS-SPME-PTV-GC-MS	IF, THM_4_	87.0–103.0	0.0010–0.020(IF:0.0010)	1.0–23.0	12.0–16.0	High	High	[20]
MLLE-PTV-GC-MS	THM_4_	93.0–99.0	0.018–0.060	5.6–6.4	6.3–7.4	High	High	[45]
HS-LPME-GC-MS	THM_4_	–	0.42–0.78	8.0–11.6	–	High	High	[46]

LLE: Liquid-liquid extraction; SPE: Solid phase extraction; SPME: Solid phase micro-extraction; SDME: Single drop micro-extraction; DLLME: Dispersive liquid–liquid micro-extraction; HFLPME :Hollow fiber liquid-phase micro-extraction; MLLE: Micro liquid–liquid extraction; LPME: Liquid phase micro-extraction; PTV: A programmed temperature evaporizer inlet; HS: Headspace; IS-PCR-IC: A post-column reaction-ion chromatography analyzer (PCR-IC) with automated internal standardization (IS); PAEKI-CE-MS/MS: Pressure-assisted electrokinetic injection for on-line enrichment in capillary electrophoresis–mass spectrometry; GC-ECD: Gas chromatography coupled with electron capture detector; GC-MS: Gas chromatography coupled with mass spectrometry; LC-MS/MS: Liquid chromatography tandem mass spectrometry; HPLC-MS/MS: High performance liquid chromatography tandem mass spectrometry; UPLC-MS/MS: Ultra-performance liquid chromatography tandem mass spectrometry. THM_4_ include CF, BDCM, CDBM and BF; HAA_5_ include CAA, BAA, DCAA, DBAA and TCAA; HAA_6_ include CAA, BAA, DCAA, DBAA, TCAA and BCAA; HAA_9_ include CAA, BAA, DCAA, DBAA, TCAA, BCAA, BDCAA, CDBAA and TBAA.

### Application of the New LLE-GC-ECD Method to Field Samples

**Figure 7:**
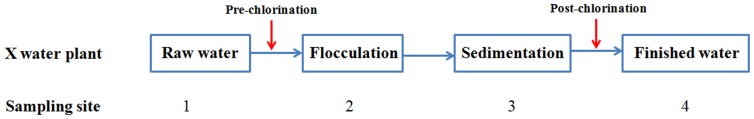
Treatment processes of the drinking water treatment plant and sampling sites.

In order to validate the optimized method that was developed in this study, drinking water samples were collected and analyzed for the target DBP compounds. Fig. 7 shows the sampling sites and chlorination points from the chosen Chinese drinking water treatment plant. The characteristics of the raw water were as follows: the temperature was 29°C, the pH was 7.8, and the concentration of NH_3_-N was 0.6 mg/L. The chloride concentration was19 mg/L, and the concentrations of bromide and iodide in the raw water were 30.00 and 4.32 µg/L, respectively. Dissolved oxygen and chemical oxygen demand were 5.03 and 2.16 mg/L respectively. The total residual chlorine was 0.27 mg/L.

Table 6 summarizes the results obtained for n = 3. IF, BF, CAA, BAA and TBAA were not detected in these samples and IAA was in the range of 0.13–0.41 µg/L in raw water (lowest) and treated waters (highest). We did not detect IF in samples collected in summer. DCAA was the HAA measured at the highest concentration (9.42 µg/L) while the concentrations of other HAAs were between non-detectable levels and 6.22 µg/L. CF had the highest concentration (6.22–9.93 µg/L) among the four THMs while BDCM and CDBM ranged from 0.26 µg/L to 3.11 µg/L.

**Table 6:** Mean concentrations of IAA, HAA_9_ and IF, THM_4_ in field water samples collected from a water treatment plant (µg/L, n = 3).

Compound	Raw water	Flocculated water	Settled water	Finished drinking water*
IAA	0.13	0.47	0.38	0.41
CAA	ND	ND	ND	ND
BAA	ND	ND	ND	ND
DCAA	0.78	7.18	9.42	6.71
BCAA	0.78	4.12	6.22	5.63
DBAA	ND	0.092	0.30	0.014
TCAA	ND	1.96	2.80	1.80
BDCAA	ND	1.96	3.38	2.98
CDBAA	ND	0.36	0.58	0.51
TBAA	ND	ND	ND	ND
IF	ND	ND	ND	ND
CF	6.22	7.39	9.74	9.93
BDCM	0.65	1.96	3.11	2.83
CDBM	0.26	0.85	1.26	1.10
BF	ND	ND	ND	ND

ND: not detected.

* Finished drinking water means the water collected from a point just before leaving the plant.

### Conclusions

A rigorous statistical approach, applying factorial and Doehlert design, was used to optimize LLE GC-ECD method conditions to achieve the simultaneous detection of iodoform and iodoacetic acid alongside regulated trihalomethanes and haloacetic acids in drinking water. The new method achieves low detection limits, high recovery and sensitivity for the target analytes. The method was validated using water samples collected from a Chinese drinking water treatment plant.

## Supporting Information

Table S1 **Doehlert’s experimental matrix for extraction time and anhydrous sodium sulfate of IF and THM_4_.**
(DOCX)

Table S2 **Doehlert’s experimental matrix for extraction time and anhydrous sodium sulfate of IAA and HAA_9_.**
(DOCX)

Table S3 **Doehlert’s experimental matrix for derivatization time and temperature of IAA and HAA_9_.**
(DOCX)

Table S4 **Doehlert’s experimental matrix for five variables and the corresponding experimental conditions of IAA and HAA_9_.**
(DOCX)

Table S5 **Model and 3D response surface for HAA_9_ in optimization of derivatization time and temperature.**
(DOCX)

Table S6 **Model and 3D surface response for HAA_9_ in optimization of derivatization-related reagents.**
(DOCX)
