# Supplementary material for: An Optimized Analytical Method for the Simultaneous Detection of Iodoform, Iodoacetic Acid, and Other Trihalomethanes and Haloacetic Acids in Drinking Water
Source: PLoS One. 2013 Apr 16;8(4):e60858. doi: 10.1371/journal.pone.0060858 (PMC3628783; doi:10.1371/journal.pone.0060858)
Supplement: Table S1 — Doehlert’s experimental matrix for extraction time and anhydrous sodium sulfate of IF and THM4. (DOCX) [file pone.0060858.s001.docx]

**Table S1 Doehlert’s experimental matrix for extraction time and anhydrous sodium sulfate of IF and THM_4_**

| No.  exp. | Coded values | | Real values | |
| --- | --- | --- | --- | --- |
|  | A | B | X_1_ | X_2_ |
| 1 | 0 | 0 | 13.0 | 10.0 |
| 2 | 0 | 0 | 13.0 | 10.0 |
| 3 | 0 | 0 | 13.0 | 10.0 |
| 4 | 0 | 0 | 13.0 | 10.0 |
| 5 | 0 | 0 | 13.0 | 10.0 |
| 6 | 1 | 0 | 22.0 | 10.0 |
| 7 | 0.5 | 0.866 | 17.5 | 18.0 |
| 8 | -1 | 0 | 4.0 | 10.0 |
| 9 | -0.5 | -0.866 | 8.5 | 2.0 |
| 10 | 0.5 | -0.866 | 17.5 | 2.0 |
| 11 | -0.5 | 0.866 | 8.5 | 18.0 |

A and X_1_: Amount of anhydrous sodium sulfate and unit is g;

B and X_2_: Extraction time and unit is min.
