# Supplementary material for: An Optimized Analytical Method for the Simultaneous Detection of Iodoform, Iodoacetic Acid, and Other Trihalomethanes and Haloacetic Acids in Drinking Water
Source: PLoS One. 2013 Apr 16;8(4):e60858. doi: 10.1371/journal.pone.0060858 (PMC3628783; doi:10.1371/journal.pone.0060858)
Supplement: Table S2 — Doehlert’s experimental matrix for extraction time and anhydrous sodium sulfate of IAA and HAA9. (DOCX) [file pone.0060858.s002.docx]

**Table S2 Doehlert’s experimental matrix for extraction time and anhydrous sodium sulfate of IAA and HAA_9_**

| No.  exp. | Coded values | | Real values | |
| --- | --- | --- | --- | --- |
|  | A | B | X_1_ | X_2_ |
| 1 | 0 | 0 | 18.0 | 10.0 |
| 2 | 0 | 0 | 18.0 | 10.0 |
| 3 | 0 | 0 | 18.0 | 10.0 |
| 4 | 0 | 1 | 18.0 | 18.0 |
| 5 | 0.866 | 0.5 | 26.0 | 14.0 |
| 6 | 0 | -1 | 18.0 | 2.0 |
| 7 | -0.866 | -0.5 | 10.0 | 6.0 |
| 8 | -0.866 | 0.5 | 10.0 | 14.0 |
| 9 | 0.866 | -0.5 | 26.0 | 6.0 |

A and X_1_: Amount of anhydrous sodium sulfate and unit is g;

B and X_2_: Extraction time and unit is min.
