# Supplementary material for: An Optimized Analytical Method for the Simultaneous Detection of Iodoform, Iodoacetic Acid, and Other Trihalomethanes and Haloacetic Acids in Drinking Water
Source: PLoS One. 2013 Apr 16;8(4):e60858. doi: 10.1371/journal.pone.0060858 (PMC3628783; doi:10.1371/journal.pone.0060858)
Supplement: Table S4 — Doehlert’s experimental matrix for five variables and the corresponding experimental conditions of IAA and HAA9. (DOCX) [file pone.0060858.s004.docx]

**Table S4 Doehlert’s experimental matrix for five variables and the corresponding experimental conditions of IAA and HAA_9_**

| No.  exp. | Coded values | | | | | Real values | | | | |  |
| --- | --- | --- | --- | --- | --- | --- | --- | --- | --- | --- | --- |
|  | A | B | C | D | E | X_1_ | X_2_ | X_3_ | X_4_ | X_5_ | |
| 1 | 0 | 0 | 0 | 0 | 0 | 10.0 | 3.0 | 150 | 7.0 | 1.0 | |
| 2 | 0 | 0 | 0 | 0 | 0 | 10.0 | 3.0 | 150 | 7.0 | 1.0 | |
| 3 | 0 | 0 | 0 | 0 | 0 | 10.0 | 3.0 | 150 | 7.0 | 1.0 | |
| 4 | 0 | 0 | 0 | 0 | 0 | 10.0 | 3.0 | 150 | 7.0 | 1.0 | |
| 5 | 1 | 0 | 0 | 0 | 0 | 15.0 | 3.0 | 150 | 7.0 | 1.0 | |
| 6 | 0.5 | 0.866 | 0 | 0 | 0 | 12.5 | 5.0 | 150 | 7.0 | 1.0 | |
| 7 | 0.5 | 0.289 | 0.817 | 0 | 0 | 12.5 | 3.7 | 200 | 7.0 | 1.0 | |
| 8 | 0.5 | 0.289 | 0.204 | 0.791 | 0 | 12.5 | 3.7 | 162 | 9.0 | 1.0 | |
| 9 | 0.5 | 0.289 | 0.204 | 0.158 | 0.775 | 12.5 | 3.7 | 162 | 7.4 | 1.5 | |
| 10 | -1 | 0 | 0 | 0 | 0 | 5.0 | 3.0 | 150 | 7.0 | 1.0 | |
| 11 | -0.5 | -0.866 | 0 | 0 | 0 | 7.5 | 1.0 | 150 | 7.0 | 1.0 | |
| 12 | -0.5 | -0.289 | -0.817 | 0 | 0 | 7.5 | 2.3 | 100 | 7.0 | 1.0 | |
| 13 | -0.5 | -0.289 | -0.204 | -0.791 | 0 | 7.5 | 2.3 | 138 | 5.0 | 1.0 | |
| 14 | -0.5 | -0.866 | -0.204 | -0.158 | -0.775 | 7.5 | 1.0 | 138 | 6.6 | 0.5 | |
| 15 | 0.5 | -0.289 | 0 | 0 | 0 | 12.5 | 2.3 | 150 | 7.0 | 1.0 | |
| 16 | 0.5 | -0.289 | -0.817 | 0 | 0 | 12.5 | 2.3 | 100 | 7.0 | 1.0 | |
| 17 | 0.5 | -0.289 | -0.204 | -0.791 | 0 | 12.5 | 2.3 | 138 | 5.0 | 1.0 | |
| 18 | 0.5 | -0.289 | -0.204 | -0.158 | -0.775 | 12.5 | 2.3 | 138 | 6.6 | 0.5 | |
| 19 | -0.5 | 0.866 | 0 | 0 | 0 | 7.5 | 5.0 | 150 | 7.0 | 1.0 | |
| 20 | 0 | 0.577 | -0.817 | 0 | 0 | 10.0 | 4.3 | 100 | 7.0 | 1.0 | |
| 21 | 0 | 0.577 | -0.204 | -0.791 | 0 | 10.0 | 4.3 | 138 | 5.0 | 1.0 | |
| 22 | 0 | 0.577 | -0.204 | -0.158 | -0.775 | 10.0 | 4.3 | 138 | 6.6 | 0.5 | |
| 23 | -0.5 | 0.289 | 0.817 | 0 | 0 | 7.5 | 3.7 | 200 | 7.0 | 1.0 | |
| 24 | 0 | -0.577 | 0.817 | 0 | 0 | 10.0 | 1.7 | 200 | 7.0 | 1.0 | |
| 25 | 0 | 0 | 0.613 | -0.791 | 0 | 10.0 | 3.0 | 188 | 5.0 | 1.0 | |
| 26 | 0 | 0 | 0.613 | -0.158 | -0.775 | 10.0 | 3.0 | 188 | 6.6 | 0.5 | |
| 27 | -0.5 | 0.289 | 0.204 | 0.791 | 0 | 7.5 | 3.7 | 162 | 9.0 | 1.0 | |
| 28 | 0 | -0.577 | 0.204 | 0.791 | 0 | 10.0 | 1.7 | 162 | 9.0 | 1.0 | |
| 29 | 0 | 0 | -0.613 | 0.791 | 0 | 10.0 | 3.0 | 112 | 9.0 | 1.0 | |
| 30 | 0 | 0 | 0 | 0.633 | -0.775 | 10.0 | 3.0 | 150 | 8.6 | 0.5 | |
| 31 | -0.5 | 0.289 | 0.204 | 0.158 | 0.775 | 7.5 | 3.7 | 162 | 7.4 | 1.5 | |
| 32 | 0 | -0.577 | 0.204 | 0.158 | 0.775 | 10.0 | 1.7 | 162 | 7.4 | 1.5 | |
| 33 | 0 | 0 | -0.613 | 0.158 | 0.775 | 10.0 | 3.0 | 112 | 7.4 | 1.5 | |
| 34 | 0 | 0 | 0 | -0.633 | 0.775 | 10.0 | 3.0 | 150 | 5.4 | 1.5 | |

A and X_1_: Concentration of Acidic methanol and unit is %;

B and X_2_: Volume of Acidic methanol and unit is mL;

C and X_3_: Concentration of Na2SO4 solution and unit is g/L;

D and X_4_: Volume of Na2SO4 solution and unit is mL;

E and X_5_: Volume of saturated NaHCO3 solution and unit is mL.
