# Supplementary material for: An Optimized Analytical Method for the Simultaneous Detection of Iodoform, Iodoacetic Acid, and Other Trihalomethanes and Haloacetic Acids in Drinking Water
Source: PLoS One. 2013 Apr 16;8(4):e60858. doi: 10.1371/journal.pone.0060858 (PMC3628783; doi:10.1371/journal.pone.0060858)
Supplement: Table S5 — Model and 3D response surface for HAA9 in optimization of derivatization time and temperature. (DOCX) [file pone.0060858.s005.docx]

**Table S5 Model and 3D response surface for HAA_9_ in optimization of derivatization time and temperature**

| Compound | Model  (Second-order model) | 3D response surface |
| --- | --- | --- |
| CAA | *Y*= +37230.39  -1316.29*X_1_*  -18386.85*X_2_*  +1262.91*X_1_X_2_*  -2262.68*X_1_^2^*  -17774.06*X_2_^2^* | 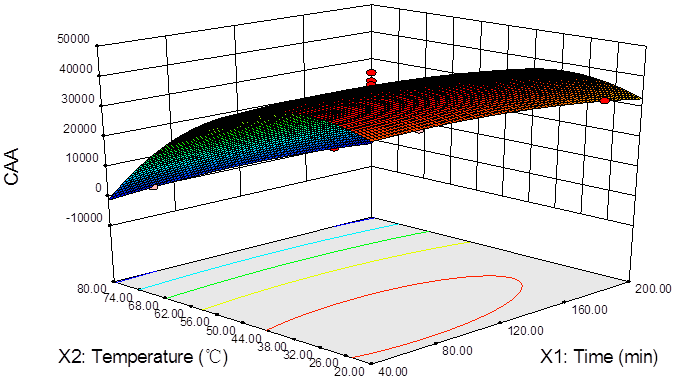 |
| BAA | *Y*= +3.775E+005  -1.882E+005*X_2_*  -1.893 E+005*X_2_^2^* | 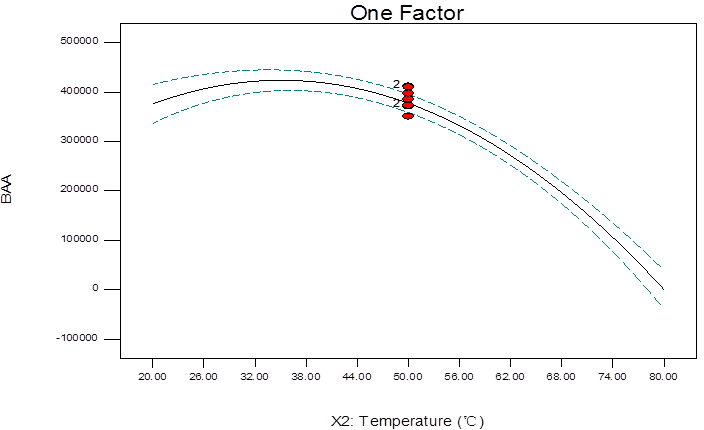 |
| DCAA | *Y*= +3.688E+005  +13192.21*X_1_*  -1.466E+005*X_2_*  -16995.67*X_1_X_2_*  -26975.89*X_1_^2^*  -2.095E+005*X_2_^2^* | 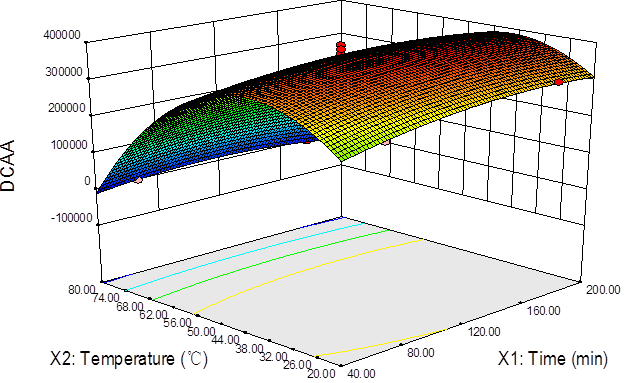 |

**Table S5 (Continued)**

| Compound | Model  (Second-order model) | 3D response surface |
| --- | --- | --- |
| BCAA | *Y*= +8.219E+005  +1.242E+005*X_1_*  -1.572E+005*X_2_*  -50815.75*X_1_X_2_*  -1.454E+005*X_1_^2^*  -5.960E+005*X_2_^2^* | 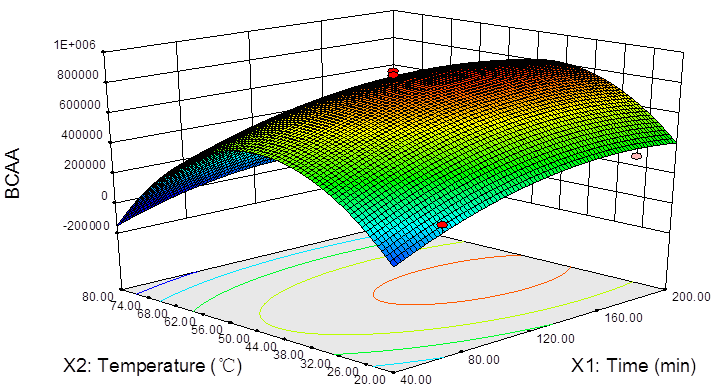 |
| DBAA | *Y*= +6.736E+005  +76746.47*X_1_*  -2.020E+005*X_2_*  -57235.42*X_1_X_2_*  -63223.18*X_1_^2^*  -4.417E+005*X_2_^2^* | 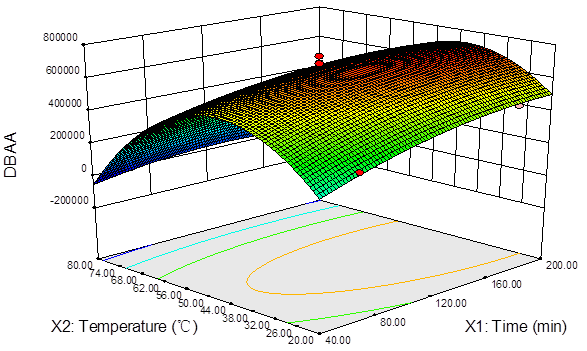 |
| TCAA | *Y*= +6.127E+005  +47000.73*X_1_*  -2.357E+005*X_2_*  -45954.76*X_1_X_2_*  -34820.52*X_1_^2^*  -3.605E+005*X_2_^2^* | 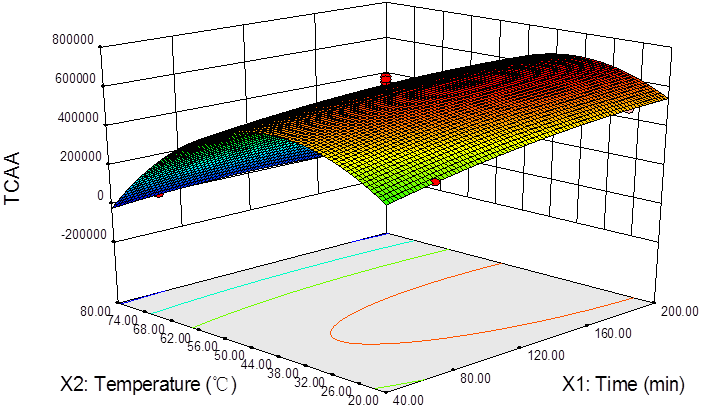 |

**Table S5 (Continued)**

| Compound | Model  (Second-order model) | 3D response surface |
| --- | --- | --- |
| BDCAA | *Y*= +1.935E+005  +91122.62*X_1_*  -94776.65*X_2_*  -39413.75*X_1_X_2_*  +66151.79*X_1_^2^*  -1.300E+005*X_2_^2^* | 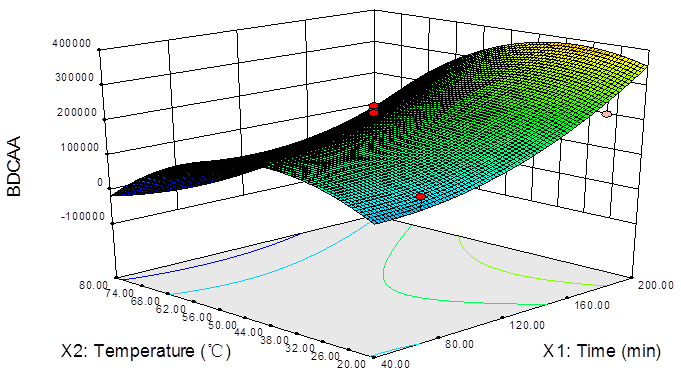 |
| CDBAA | *Y*= +2.490E+005  +70797.94*X_1_*  -44790.40*X_2_*  -23450.11*X_1_X_2_*  -2.042E+005*X_2_^2^* | 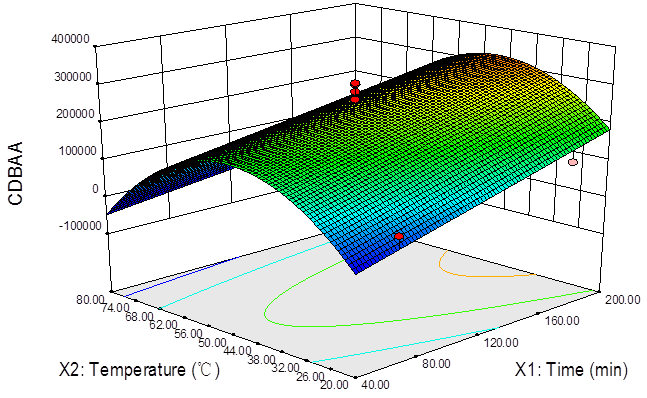 |
| TBAA | *Y*= +1.696E+005  +37096.15*X_1_*  -22232.85*X_2_*  -12423.78*X_1_X_2_*  -24782.45*X_1_^2^*  -1.356E+005*X_2_^2^* | 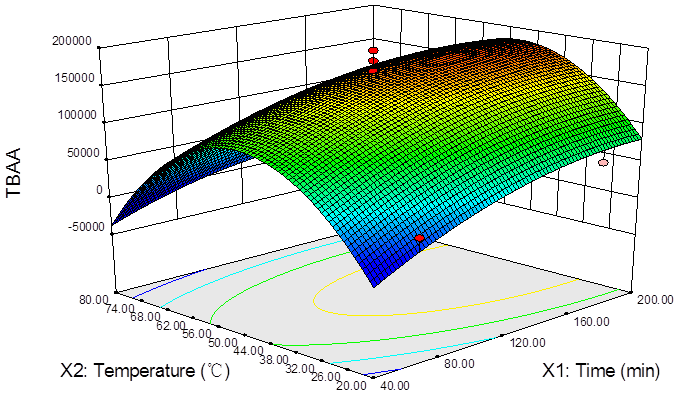 |

*Y* is the peak area of HAA_9_ *, X*_1_ is derivatization time (min), *X*_2_ is derivatization temperature (°C)
