# Supplementary material for: An Optimized Analytical Method for the Simultaneous Detection of Iodoform, Iodoacetic Acid, and Other Trihalomethanes and Haloacetic Acids in Drinking Water
Source: PLoS One. 2013 Apr 16;8(4):e60858. doi: 10.1371/journal.pone.0060858 (PMC3628783; doi:10.1371/journal.pone.0060858)
Supplement: Table S6 — Model and 3D surface response for HAA9 in optimization of derivatization-related reagents. (DOCX) [file pone.0060858.s006.docx]

**Table S6 Model and 3D surface response for HAA_9_ in optimization of derivatization-related reagents**

| Compound | Model* | 3D surface response** |
| --- | --- | --- |
| CAA | *Y*= +32439.15  -13005.92*X_2_* | 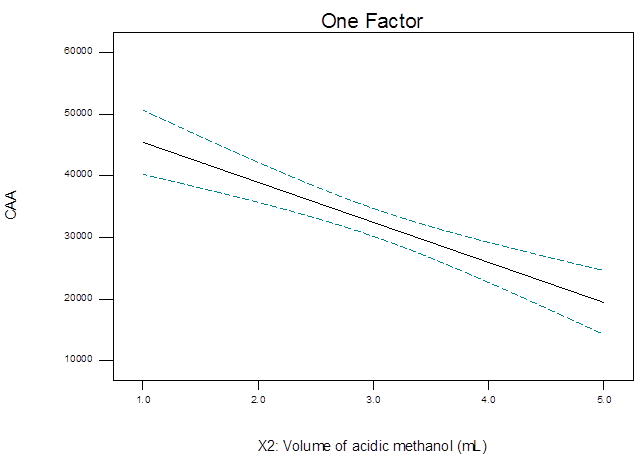 |
| BAA | *Y*= +4.69E+005  -1.00E+005*X_2_* | 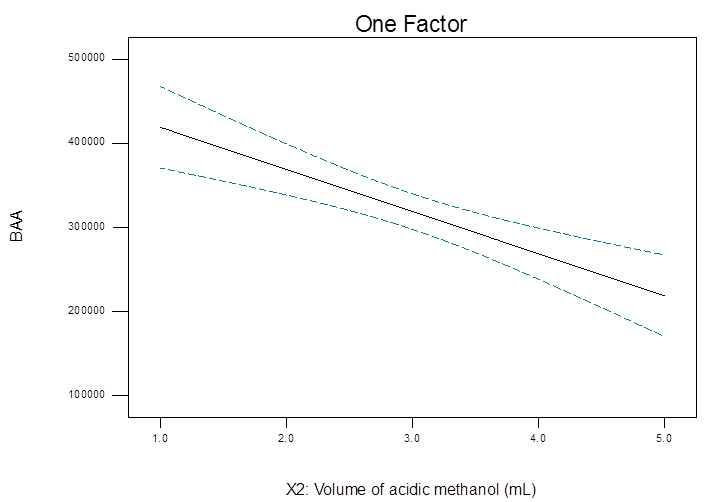 |
| DCAA | *Y=*  +3.11E+005  +52929.96*X_1_*  -62744.57*X_2_* | 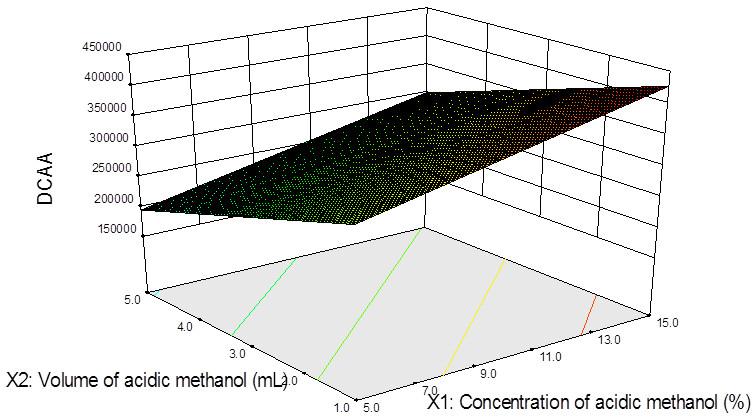 |

**Table S6 (Continued)**

| Compound | Model* | 3D surface response** |
| --- | --- | --- |
| BCAA | *Y*= +6.83E+005  +1.58E+005*X_1_* | 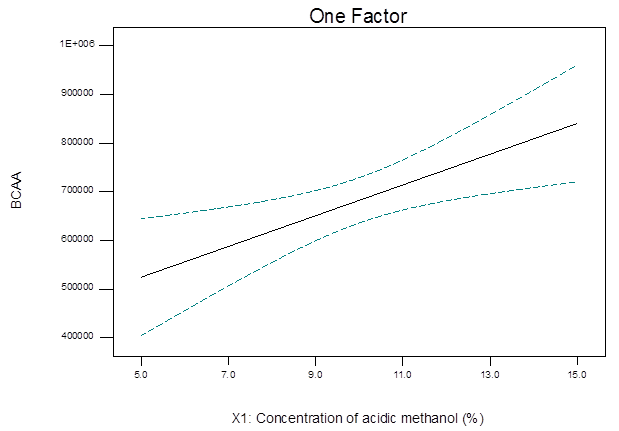 |
| DBAA | *Y*= +2.91E+005  +1.24E+005*X_1_*  -1.47E+005*X_2_*  -53169.35X_3_  +48047.64X_4_  +7.16E+005X_5_  -1.72E+005*X_1_X_2_*  +2.47E+005*X_1_*X_3_  +1.85E+005*X_2_X_4_*  -1.23E+005*X_3_^2^*  -82426.62*X_4_^2^*  -3.17E+005*X_5_^2^* | 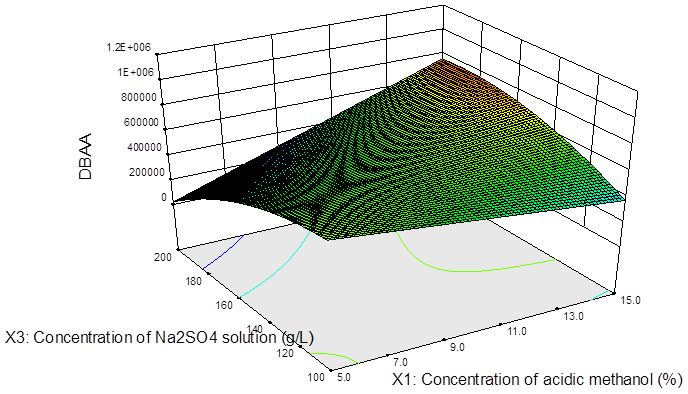 |
| TCAA | *Y*= +2.24E+005  +93259.47*X_1_*  -1.30E+005*X_2_*  -36412.25X_3_  +41993.10X_4_  +6.91E+005X_5_  +1.65E+005*X_1_*X_3_  +1.53E+005*X_2_X_4_*  -1.03E+005*X_3_^2^*  *-*70397.76*X_4_^2^*  *-*3.01E+00*5X_5_^2^* | 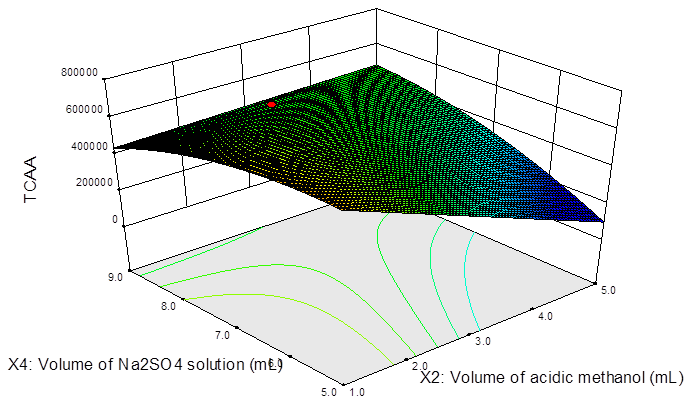 |

**Table S6 (Continued)**

| Compound | Model* | 3D surface response** |
| --- | --- | --- |
| BDCAA | *Y*= +1.07E+005  -89543.62*X_3_*  +1.04E+005*X_4_*  +2.35E+005*X_5_* | 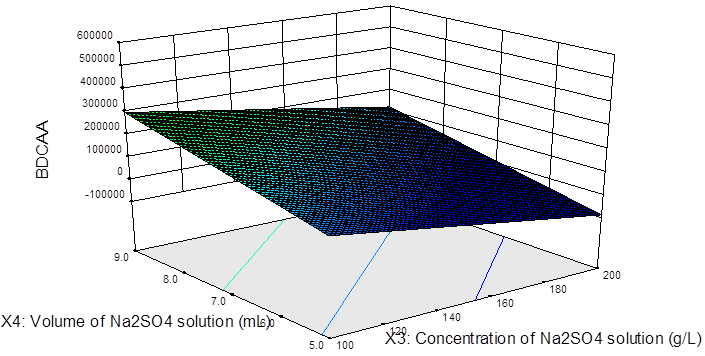 |
| CDBAA | *Y*= +1.11E+005  +22237.93*X_1_*  -7951.05*X_2_*  -62097.07X_3_  +76422.93X_4_  +1.36E+005X_5_  -1.10E+005*X_1_*X_2_  +1.08E+005*X_1_X_3_*  90651.07*X_3_X_4_* | 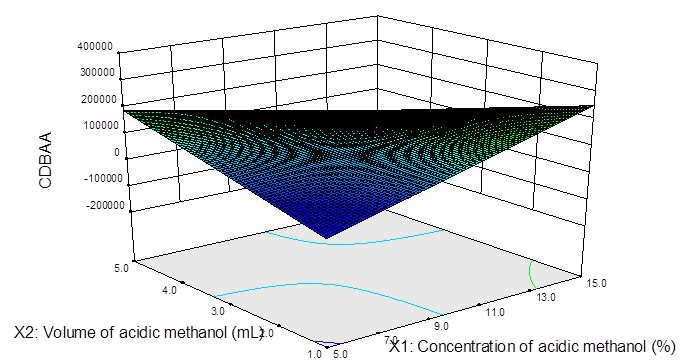 |
| TBAA | *Y*= +69338.52  -26803.06*X_3_*  +35246.50*X_4_*  +40645.67*X_5_* | 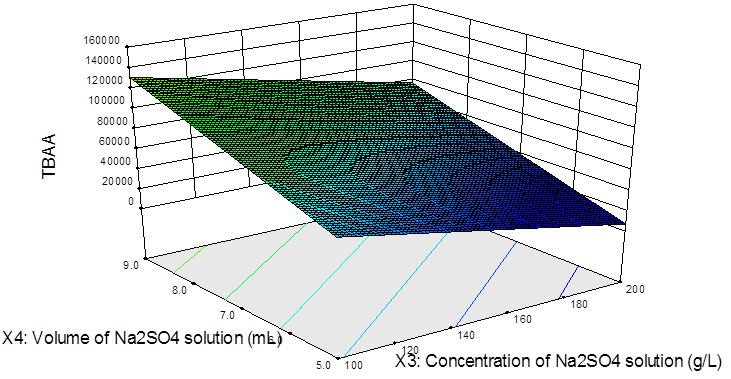 |

*: *Y* is the peak area of HAA_9_; X_1_ is the concentration of acidic methanol (%); X_2_ is the volume of Acidic methanol (mL); X_3_ is the concentration of Na_2_SO_4_ solution (g/L); X_4_ is the volume of Na_2_SO_4_ solution (mL); X_5_ is the volume of saturated NaHCO_3_ solution (mL).

**: Only provided 3D response surface of one or two factors which had the most significant impact statistically and not included the factor of the volume of saturated NaHCO_3_ solution.
